# Mental health and ecological momentary assessments during COVID-19: Data from the corona health app adolescents study

**DOI:** 10.1016/j.dib.2025.111619

**Published:** 2025-05-07

**Authors:** Michael Winter, Lorenz Deserno, Marcel Romanos, Rüdiger Pryss

**Affiliations:** a Institute of Medical Data Science, University Hospital Würzburg, Würzburg, Germany; b Institute of Clinical Epidemiology and Biometry, University of Würzburg, Würzburg, Germany; c Center of Mental Health, Department for Child and Adolescent Psychiatry, Psychosomatics and Psychotherapy, University Hospital of Würzburg, Würzburg, Germany

**Keywords:** COVID-19, Ecological momentary assessment, Public health surveillance, Digital health monitoring

## Abstract

The dataset presented in this work is derived from the Adolescent Study in the Corona Health app, a digital health initiative designed with the German Robert Koch Institute (RKI) to monitor the physical and mental well-being of children during and after the COVID-19 pandemic. Data were collected using a mobile-based survey platform that utilized Ecological Momentary Assessments (EMA) and Patient-Reported Outcome Measures (PROMs) to capture real-time health-related information. The dataset comprises responses from a single longitudinal study involving 399 adolescents between 12 and 17 years who completed baseline assessments, with 113 of these participants also contributing 637 follow-up assessments over time. The study utilized baseline and follow-up questionnaires to capture changes in participants' health status throughout the pandemic period (July 2020 to December 2023). The questionnaires cover key health indicators such as medical history, lifestyle factors, emotional well-being, and pandemic-related behavioral changes. By capturing real-time, longitudinal mental health data from adolescents during a significant public health crisis, this dataset enables researchers to examine how anxiety, depression, and quality-of-life indicators fluctuated in response to pandemic restrictions. The integration of ecological momentary assessments with validated psychological screening tools provides unique opportunities to analyze adolescent mental health trajectories, identify resilience factors, and evaluate the effectiveness of mobile health approaches for public health surveillance during crisis situations. The data, including questionnaire responses and mobile sensing data, are publicly available under a Creative Commons license at https://zenodo.org/records/15101756.

Specifications Table

**Table:** 

Subject	Health Sciences, Medical Sciences & Pharmacology
Specific subject area	Smartphone-based mental health assessments of adolescent anxiety and depression during COVID-19.
Type of data	Table, Fig., Raw
Data collection	The Corona Health app was used to collect data through self-reported surveys, incorporating Ecological Momentary Assessments (EMA) and Patient-Reported Outcome Measures (PROMs). The app recorded user input via structured questionnaires and passive sensor data, including app usage statistics. Questionnaires were derived from validated instruments and translated into eight languages.
Data source location	Data was collected worldwide, most of them from Germany. Data source location is the Institute of Clinical Epidemiology and Biometry, University of Würzburg, Würzburg, Germany (49.7882° N, 9.9353° E)
Data accessibility	Repository name: Zenodo DOI: 10.5281/zenodo.15101756 Direct URL to data: https://zenodo.org/records/15101756
Related research article	M. Weiß, et al., Common and differential variables of anxiety and depression in adolescence: a nation-wide smartphone-based survey. *Child and Adolescent Psychiatry and Mental Health,* 18(1) (2024), 103 https://link.springer.com/article/10.1186/s13034-024-00793-1

## 1 Value of the Data

• The data collected in the Corona Health Adolescent Study provide valuable insights into the mental health and well-being of adolescents, particularly in the context of the COVID-19 pandemic and its aftermath from July 2020 until December 2023 [1]. Utilizing Ecological Momentary Assessment (EMA) and Patient-Reported Outcome Measures (PROMs), this dataset captures real-time self-reports on symptoms of anxiety, depression, and overall emotional well-being. By integrating baseline and follow-up assessments, the data enable the study of mental health trajectories and risk factors (e.g., depression, anxiety, alcohol abuse) affecting adolescents over time [2,3].
• This dataset allows for an in-depth analysis of behavioral, social, and psychological influences on adolescent mental health. It includes a broad spectrum of variables, such as mental health, quality of life, family dynamics, and COVID-19 pandemic-related concerns, offering a multifaceted perspective on adolescent well-being (e.g., lifestyle habids and pandemic-related stressors). The study’s smartphone-based methodology ensures a high ecological validity, reducing recall bias, and increasing accessibility for participants who might be less likely to engage in traditional mental health assessments.
• While the dataset is based on a convenience sample, its comprehensive scope and methodological rigor provide valuable opportunities for exploring patterns of adolescent mental health challenges and resilience factors. This was ensured by relying on validated instruments, multilingual availability, and standardized digital protocols. The structured data collection process, ethical compliance, and use of validated psychological screening tools enhance its applicability for mental health research, early intervention strategies, and public health initiatives. This dataset serves as an important resource for advancing knowledge on adolescent mental health, informing preventive programs, digital health interventions, and evidence-based policy recommendations.
• This dataset offers opportunities for longitudinal analyses of adolescent mental health trajectories during and after pandemic restrictions, as it spans from July 2020 to December 2023. Researchers can examine how anxiety, depression, and quality-of-life indicators fluctuated in response to changing pandemic policies, school closures, and social isolation measures. The inclusion of follow-up data from 113 participants allows for the investigation of resilience factors and adaptation patterns over time, providing insights into which adolescents recovered quickly versus those who experienced prolonged mental health effects.
• The dataset's comprehensive documentation and structured format enable diverse methodological approaches, supporting methods ranging from statistical modelling to exploratory machine learning approaches, and integration with other COVID-19 mental health datasets. Researchers can leverage the standardized assessment tools (validated anxiety and depression measures) to conduct comparative analyses with regional or international adolescent mental health data. Additionally, the combination of self-reported questionnaires and digital phenotyping offers unique possibilities for examining relationships between subjective experiences and objective behavioral patterns (e.g., correlating self-reported anxiety levels with app usage patterns or location mobility, potentially informing the development of more responsive digital mental health interventions for adolescents.

## 2 Background

During the initial phase of the COVID-19 pandemic in 2020, we partnered with the German Robert Koch Institute to develop the Corona Health smartphone application [1] Corona Health constituted a digital platform aimed at capturing the physical and mental health impacts of the COVID-19 pandemic on the general population. This app enabled public participation in studies tracking mental and physical health throughout the pandemic, gathering real-time ecological data on how pandemic-related factors impacted individual well-being.

Corona Health combines several methodologies: Ecological Momentary Assessments (EMA) [4], Patient-Reported Outcome Measures (PROMs) [5], Mobile Crowdsensing [6], and Digital Phenotyping [7]. These approaches collect high-resolution, real-time data that overcomes limitations of conventional retrospective surveys, which often suffer from recall bias and fail to capture dynamic changes in mental states and behaviors.

The app allows users to participate in baseline and follow-up assessments, enabling longitudinal analysis of health trends. With consent, it also records GPS location data and digital phenotyping metrics. Our dataset focuses on adolescent mental health, including 399 baseline participants aged 12 - 17 who contributed 637 follow-up questionnaire responses between July 2020 and December 2023. Table 1 presents a concise overview of demographics.

**Table 1:** Demographics and key numbers about the baseline questionnaire.

No. Questionnaires	399 – Baseline 637 - Follow-up - 113 users
No. Tracking GPS	214 (53.63%)
No. Tracking App Usage	38 (9.52%)
mobile OS	325 (81.50%) – Android 74 (18.50%) - iOS
Age (mean (SD), in years)	15.22 (1.57)
Gender	140 (35.09%) – Male 133 (33.33%) – Female 4 (1.0%) – Divers 6 (1.5%) - Not reported 117 (29.32%) - Missing
General Health	43 (10.78%) – Excellent 75 (18.8%) - Very good 104 (26.07%) – Good 52 (13.03%) – Fair 9 (2.26%) – Poor 117 (29.32%) - Missing
In psychotherapy?	227 (56.89%) – No 14 (3.51%) - Yes, but not now 8 (2.01%) - Yes, but remotely 34 (8.52%) - Yes, regularly 117 (29.32%) - Missing

## 3 Data Description

The datasets and accompanying codebook are accessible under a Creative Commons license at https://zenodo.org/records/15101756 [11]. A comprehensive breakdown of all questionnaire items can be found in Table 2 (see at the end of the document), with the full codebook (see ch_ado.xlsx) also available on Zenodo. In addition to the processed datasets, Zenodo hosts the raw data, encompassing both baseline (see RKI_ado_baseline.csv) and follow-up (see RKI_ado_followUp.csv) questionnaires, which comprise questionnaire and sensing data, along with structured versions where baseline and follow-up data have been separated for ease of analysis.

**Table 2:** Detailed description of the baseline and follow-up questionnaire. Because these questionnaires share a major part of their questions, we provide these here within one table.

Question Type	Label	Follow-Up?	Question	Encoding and Answer Options
SingleChoice	kj_age	No	How old are you (in years)?	Integer
SingleChoice	kj_sex	No	What is your gender?	1 = Male, 2 = Female, 3 = Diverse, 4 = No Answer
FreeText	kj_nat	No	What is your nationality?	String
FreeText	kj_region	No	In which country do you live?	String
Slider	kj_house1	No	How many persons currently live in your household?	Min = 1, Max = 10, Step = 1
Slider	kj_house2	No	How many of these are children or adolescents (i.e. under the age of 18 years)?	Min = 1, Max = 9, Step = 1
MultipleChoice	kj_liv	No	Which of these describes your home? (Multiple selections possible)	1 = Apartment, 2 = House, 3 = With balcony, 4 = With terrace, 5 = With garden
SingleChoice	kj_school1	No	Are you attending school or are you pursuing other activities/occupations?	1 = School, 2 = Apprenticeship, 3 = Occupational school, 4 = University, 5 = Employed, 6 = Jobless, 7 = Other
SingleChoice	kj_school2	No	Which degree are you planning to complete (or have you completed)?	1 = I don't know, 2 = Left school without qualification, 3 = Certificate of Secondary Education, 4 = Vocational/technical diploma, 5 = University-entry diploma/A levels/final secondary-school examinations
SingleChoice	kj_school3	No	What is your opinion/feeling about being schooled at home / about working remotely from home compared to regular school/work?	1 = Much more stressful, 2 = More stressful, 3 = Not different, 4 = More pleasant, 5 = Much more pleasant
Slider	kj_ses	No	Answer this question by marking a number from 1 to 10. At ``10'' are those families with the most money, with the best education and the best jobs. At ``1'' are those families who are poorest, have poor education and the least jobs or no jobs. Now think of your family. At which number would your family be?	Min = 1, Max = 10, Step = 1
Slider	kj_life	No	Answer this question by marking a number from (0) to (10). (10) signifies the best imaginable life. (0) signifies the worst imaginable life. What number would your life currently be?	Min = 0, Max = 10, Step = 1
SingleChoice	kj_health	No	In general how would you describe your health?	1 = Excellent, 2 = Very good, 3 = Good, 4 = Fair, 5 = Poor
SingleChoice	kj_diag	No	Have you ever been told by a doctor or therapist that you have a mental illness?	0 = No, 1 = Yes, ADHD, 2 = Yes, anxiety, 3 = Yes, depression, 4 = Yes, eating disorder, 5 = Yes, autism, 6 = Yes, addiction, 7 = Yes, other, 8 = Don't know
SingleChoice	kj_psyth	No	Are you in psychotherapy?	0 = No, 1 = Yes, but not currently due to the Corona pandemic, 2 = Yes, but currently via telephone/online due to the Corona pandemic, 3 = Yes, I regularly go to psychotherapy
SingleChoice	kj_smoke	No	Do you smoke?	0 = No, I have never smoked, 1 = Yes, every day, 2 = Yes, several days per week, 3 = Yes, once a week, 4 = Yes, less than once a week
SingleChoice	kj_alc	No	Do you drink alcohol?	0 = No, I have never had alcohol, 1 = Yes, once a month or less, 2 = Yes, 2 to 4 times a month, 3 = Yes, 2 to 3 times a week, 4 = Yes, 4 times a week or more
SingleChoice	kj_adhd1	No	Do you find it difficult to focus attention on tasks (e.g. homework) or on play activities (e.g. a board game)?	1 = Never, 2 = Sometimes, but it does not cause problems, 3 = Yes, and it causes problems for me
SingleChoice	kj_adhd2	No	Are you easily distracted during tasks that require attention?	1 = Never, 2 = Sometimes, but it does not cause problems, 3 = Yes, and it causes problems for me
SingleChoice	kj_adhd3	No	Is it difficult for you to stay seated when you are expected to?	1 = Never, 2 = Sometimes, but it does not cause problems, 3 = Yes, and it causes problems for me
SingleChoice	kj_adhd4	No	Do you act impulsively without thinking about consequences?	1 = Never, 2 = Sometimes, but it does not cause problems, 3 = Yes, and it causes problems for me
SingleChoice	kj_odd1	No	Do you easily get upset and lose your temper?	1 = Never, 2 = Sometimes, but it does not cause problems, 3 = Yes, and it causes problems for me
SingleChoice	kj_odd2	No	Do you often argue and talk back with your parents or teachers?	1 = Never, 2 = Sometimes, but it does not cause problems, 3 = Yes, and it causes problems for me
SingleChoice	kj_odd3	No	Do you defy or disobey rules at home, at school or at other places?	1 = Never, 2 = Sometimes, but it does not cause problems, 3 = Yes, and it causes problems for me
SingleChoice	kj_selfeff	No	Is the following statement true for you? If I am in trouble, I can usually think of a solution.	1 = Not at all true, 2 = Hardly true, 3 = Moderately true, 4 = Exactly true
MultipleChoice	kj_contact1	No	Since the coronavirus pandemic, how do you stay connected with your friends? You may mark more than one answer.	1 = Not at all, 2 = Via phone or audio calls, 3 = Via messaging, 4 = Via video calls, 5 = We meet in person
SingleChoice	kj_contact2	No	Since the coronavirus pandemic, how have your interactions with others outside your home changed?	1 = Much less, 2 = Somewhat less, 3 = As much, 4 = Somewhat more, 5 = Much more
YesNoSwitch	kj_contact3	No	Do you think that contact via phone or digital media can replace personal contact?	0 = No, 1 = Yes
SingleChoice	kj_media1	No	Since the coronavirus pandemic, how much time do you spend on media (e.g. TV, video games, web surfing, social media)?	1 = Much less, 2 = Somewhat less, 3 = As much as before, 4 = Somewhat more, 5 = Much more
SingleChoice	kj_famclim1	No	Since the coronavirus pandemic, how has the general mood in your family changed?	1 = Much worse, 2 = Somewhat worse, 3 = Unchanged, 4 = Somewhat better, 5 = Much better
SingleChoice	kj_famarg1	No	Since the coronavirus pandemic, how often does your family fight?	1 = Much less, 2 = Somewhat less, 3 = As much as before, 4 = Somewhat more, 5 = Much more
SingleChoice	kj_cv_inf	Yes	Have you been infected with the coronavirus (proven by a test)?	0 = No, 1 = Yes, I am currently sick, 2 = Yes, but I feel healthy (again)
YesNoSwitch	kj_cv_fam	Yes	Is somebody in your family infected with the coronavirus?	0 = No, 1 = Yes
YesNoSwitch	kj_cvad	Yes	Has a member of your family or somebody you know died due to an infection with the coronavirus?	0 = No, 1 = Yes
SingleChoice	kj_school4	Yes	Which statement is correct in regard to school/training/work?	1 = I currently attend school / go to work, 2 = I am schooled at home / I am working remotely from home, 3 = Answers do not apply
SingleChoice	kj_restr_cur	Yes	Are there currently exit or travel restrictions due to the coronavirus where you live?	0 = No, 1 = Yes, 2 = Don't know
SingleChoice	kj_restr_day	Yes	How do you feel about the restrictions due to the Corona-pandemic? Is your daily life...	1 = Much more stressful, 2 = More stressful, 3 = Unchanged, 4 = More pleasant, 5 = Much more pleasant
SingleChoice	kj_restr_out	Yes	How often do you currently go out (to school, work, take a walk, shopping, etc.)?	1 = Not at all, 2 = Once or twice a week, 3 = 3 to 4 times a week, 4 = 5 to 6 times a week, 5 = Daily
SingleChoice	kj_sport	Yes	How many times have you exercised (e.g. running, ball sports, etc.) in the past week?	1 = No sporting activity, 2 = Less than 1 hour a week, 3 = 1-2 hours a week regularly, 4 = 2-4 hours a week regularly, 5 = Regularly more than 4 hours per week
YesNoSwitch	kj_olfac	Yes	Have you experienced a loss of smell or taste?	0 = No, 1 = Yes
SingleChoice	kj_famclim2	Yes	What was the general mood in your family?	1 = Very bad, 2 = Rather bad, 3 = Moderate, 4 = Rather good, 5 = Very good
SingleChoice	kj_famarg2	Yes	Have there been fights in your family?	1 = Not at all, 2 = On 1 or 2 days, 3 = On 3 or 4 days, 4 = On 5 or 6 days, 5 = Every day
SingleChoice	kj_viol	Yes	Have you or somebody in your family experienced physical violence?	1 = Not at all, 2 = On 1 or 2 days, 3 = On 3 or 4 days, 4 = On 5 or 6 days, 5 = Every day
SingleChoice	kj_anx2	Yes	Have you worried about catching the coronavirus?	0 = Never, 1 = Sometimes, 2 = Often, 3 = Most of the time, 4 = Always
SingleChoice	kj_anx3	Yes	Have you worried about infecting someone else with the coronavirus?	0 = Never, 1 = Sometimes, 2 = Often, 3 = Most of the time, 4 = Always
SingleChoice	kj_media2	Yes	How many hours have you spent on average per day on digital media (e.g. TV, video games, web surfing, social media)?	1 = I do not use digital media at all, 2 = Less than 1 hour, 3 = 1 to 3 hours, 4 = 4 to 6 hours, 5 = More than 6 hours
SingleChoice	kj_qol1	Yes	Have you felt fit and well?	0 = Not at all, 1 = Slightly, 2 = Moderately, 3 = Very, 4 = Extremely
SingleChoice	kj_qol2	Yes	Have you felt full of energy?	0 = Never, 1 = Seldom, 2 = Quite often, 3 = Very often, 4 = Always
SingleChoice	kj_qol3	Yes	Have you felt sad?	0 = Never, 1 = Seldom, 2 = Quite often, 3 = Very often, 4 = Always
SingleChoice	kj_qol4	Yes	Have you felt lonely?	0 = Never, 1 = Seldom, 2 = Quite often, 3 = Very often, 4 = Always
SingleChoice	kj_qol5	Yes	Have you had enough time for yourself?	0 = Never, 1 = Seldom, 2 = Quite often, 3 = Very often, 4 = Always
SingleChoice	kj_qol6	Yes	Have you been able to do the things that you want to do in your free time?	0 = Never, 1 = Seldom, 2 = Quite often, 3 = Very often, 4 = Always
SingleChoice	kj_qol7	Yes	Have your parent(s) treated you fairly?	0 = Never, 1 = Seldom, 2 = Quite often, 3 = Very often, 4 = Always
SingleChoice	kj_qol8	Yes	Have you had fun with your friends?	0 = Never, 1 = Seldom, 2 = Quite often, 3 = Very often, 4 = Always
SingleChoice	kj_qol9	Yes	Have you got on well at school?	0 = Not at all, 1 = Slightly, 2 = Moderately, 3 = Very, 4 = Extremely
SingleChoice	kj_qol10	Yes	Have you been able to pay attention?	0 = Never, 1 = Seldom, 2 = Quite often, 3 = Very often, 4 = Always
SingleChoice	kj_scas1	Yes	I worry about things	1 = Never, 2 = Sometimes, 3 = Often, 4 = Always
SingleChoice	kj_scas2	Yes	I feel afraid	1 = Never, 2 = Sometimes, 3 = Often, 4 = Always
SingleChoice	kj_scas3	Yes	I worry about being away from my parents	1 = Never, 2 = Sometimes, 3 = Often, 4 = Always
SingleChoice	kj_scas4	Yes	I feel scared if I have to sleep on my own	1 = Never, 2 = Sometimes, 3 = Often, 4 = Always
SingleChoice	kj_scas5	Yes	I have trouble going to school in the mornings because I feel nervous or afraid	1 = Never, 2 = Sometimes, 3 = Often, 4 = Always
SingleChoice	kj_scas6	Yes	I suddenly start to tremble or shake when there is no reason for this	1 = Never, 2 = Sometimes, 3 = Often, 4 = Always
SingleChoice	kj_scas7	Yes	I worry that I will suddenly get a scared feeling when there is nothing to be afraid of	1 = Never, 2 = Sometimes, 3 = Often, 4 = Always
SingleChoice	kj_scas8	Yes	I would feel scared if I had to stay away from home overnight	1 = Never, 2 = Sometimes, 3 = Often, 4 = Always
SingleChoice	kj_phq_hope	Yes	Feeling down, depressed, irritable or hopeless?	0 = Not at all, 1 = Several days, 2 = More than half the days, 3 = Nearly every day
SingleChoice	kj_phq_interest	Yes	Little interest or pleasure in doing things?	0 = Not at all, 1 = Several days, 2 = More than half the days, 3 = Nearly every day
SingleChoice	kj_phq_sleep	Yes	Trouble falling asleep, staying asleep, or sleeping too much?	0 = Not at all, 1 = Several days, 2 = More than half the days, 3 = Nearly every day

### 3.1 Survey instruments and content

The adolescent study conducted through the Corona Health app includes two distinct questionnaires: a baseline survey and a follow-up survey. Additionally, with participant consent, mobile sensing data were collected to complement the self-reported responses.

### 3.2 Baseline questionnaire

The baseline questionnaire begins with a section that collects fundamental demographic details from participants, such as gender, age, and information about family and daily life. The remainder of the survey is structured into multiple sections, comprising a total of 67 questions.

• General Health and Mental Well-being: These questions capturing information about the participant's general health status, history of mental health diagnoses, therapy usage, and lifestyle habits such as smoking
• Impact of the Pandemic on Social Life and Media Usage: Questions exploring how the COVID-19 pandemic has affected participants' social interactions, changes in contact frequency, reliance on digital communication, and shifts in media consumption habits.
• Current Effects of the COVID-19 Pandemic: Questions examine the participant’s personal and family experiences with COVID-19, including infection status, impacts on daily life, school/work, travel restrictions, social behaviors, physical activity, and symptoms like loss of smell or taste.
• Emotional and Social Well-being During the Pandemic: Questions exploring the participant's emotional experiences, family dynamics, concerns about COVID-19 infection, and changes in digital media consumption over the past week.
• Well-being and Daily Life in the Past Week: Questions assessing the participant’s physical and emotional well-being, social interactions, school experiences, and overall life satisfaction over the past seven days.
• Anxiety and Emotional Well-being: Questions capturing the participant’s anxiety levels, fears, and emotional responses, including worries about separation, school-related nervousness, and sudden feelings of fear or trembling over the past seven days.
• Depressive Symptoms and Sleep Patterns: Questions evaluating the participant’s mood, interest in daily activities, and sleep disturbances over the past two weeks to assess potential signs of depression and emotional distress.

### 3.3 Follow-up questionnaire

The follow-up questionnaire examines any changes in the previously assessed topics since the participant’s last response, consisting of 36 questions in total. Generally, the follow-up questionnaire consists of a subset of questions from the baseline questionnaire (see Table 2, Column Follow-Up?).

### 3.4 Mobile sensing data

Corona Health collects mobile sensing data relevant to studying the physical and mental health of the population during the COVID-19 pandemic. This data encompasses device-related information, approximate location data recorded at the time of questionnaire completion, and aggregated application usage statistics (limited to Android devices).

### 3.5 Dataset overview

**Fig. 1:**
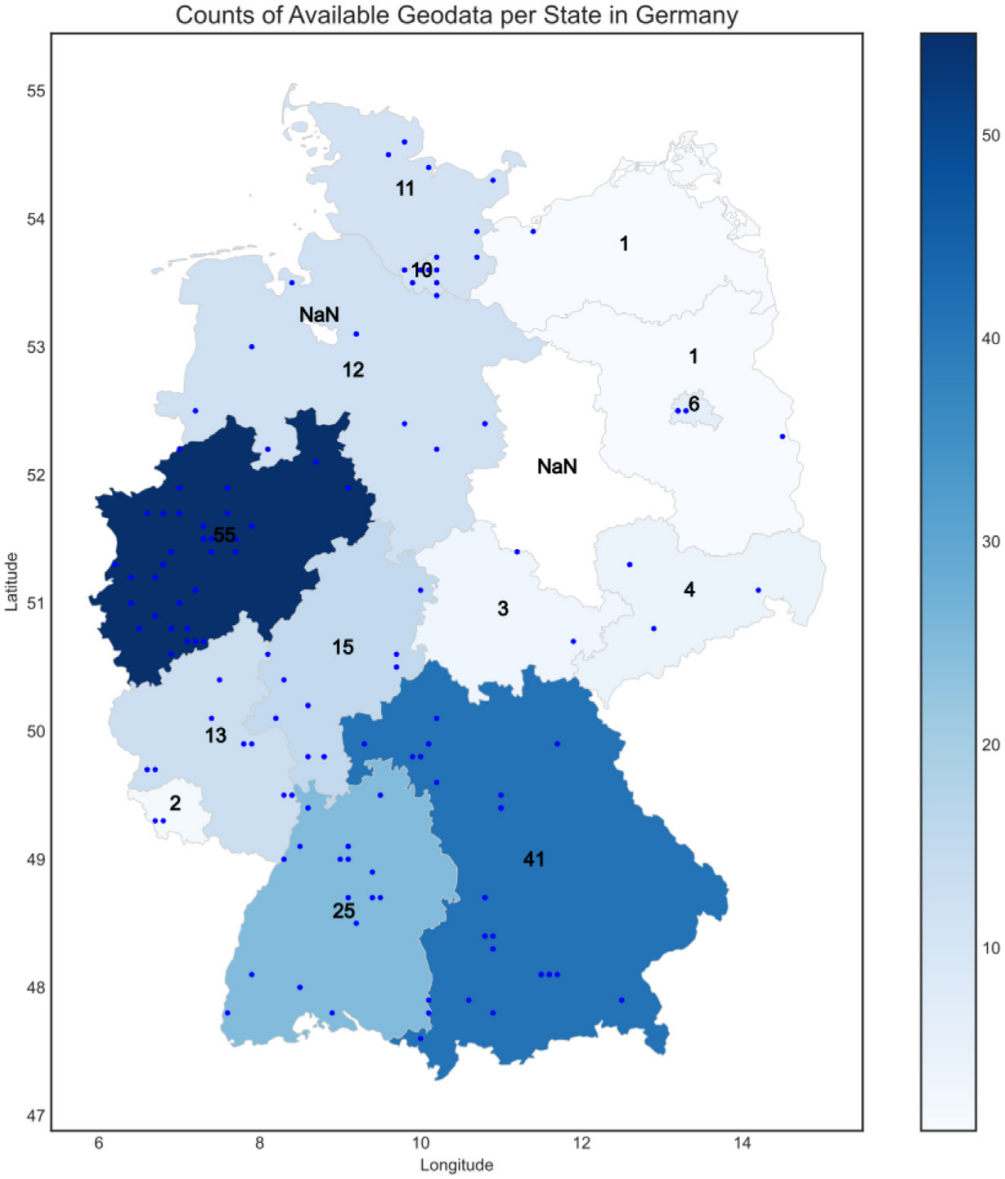
The geospatial distribution of baseline questionnaire responses in Germany (n = 199) provides an overview of participant locations at the time of completion. The heatmap visualizes sample density by state, with darker shades indicating higher concentrations of respondents.

The dataset includes responses from baseline and multiple follow-up questionnaires collected between July 2020 and December 2023. In addition, when consent was given from the participant, the data includes location data and digital phenotyping metric. The latter was limited only for Android users. More specifically, mobile OS client, Corona Health version, client device, app usage data, and longitude and latitude data are stored. Table 1 presents the baseline characteristics of the 399 participants. The gender distribution between male and female participants is relatively balanced (35.1% vs. 33.3%), with an average age of 15.22 years. 81.5% were Android and 18.5% iOS users. Additionally, 91% of participants were located in Germany at the time of baseline questionnaire completion. A visualization of the available geospatial data in Germany is depicted in Fig. 1.

**Fig. 2:**
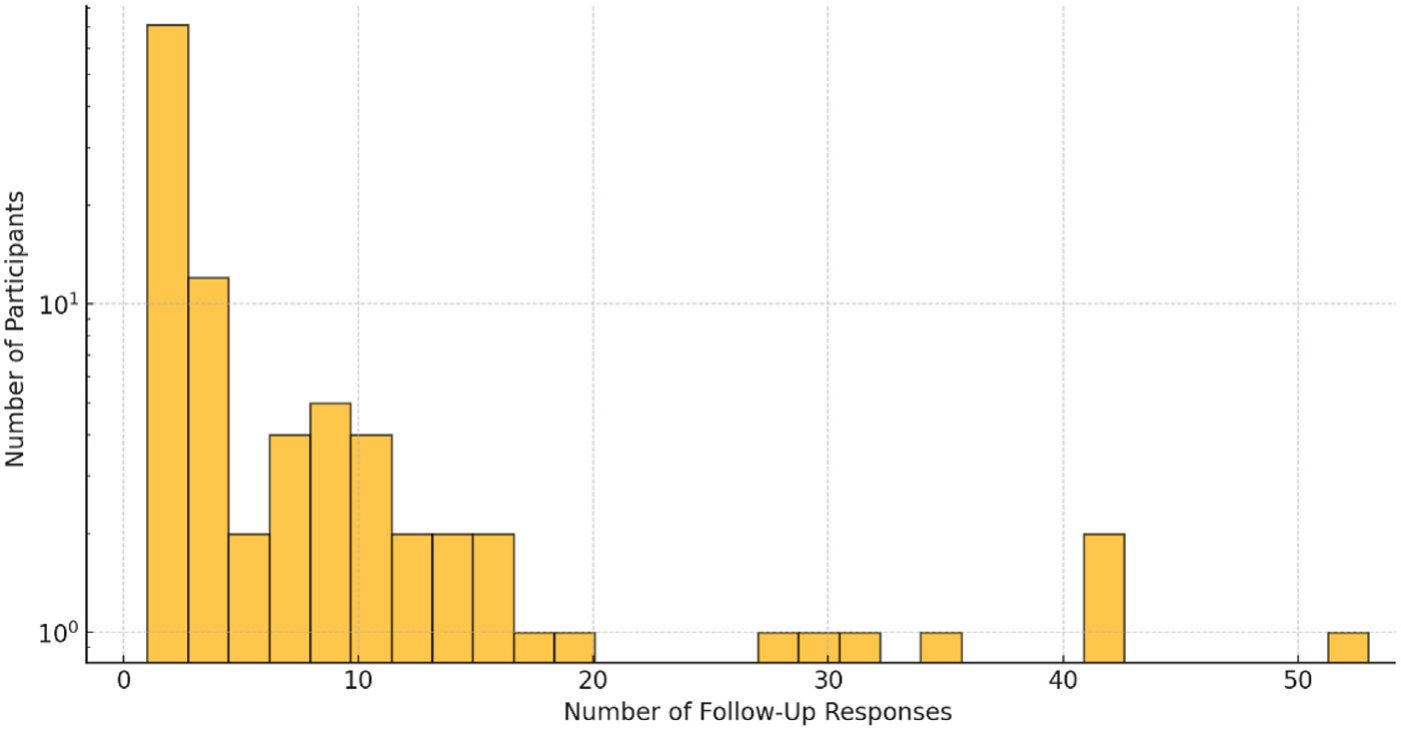
Number of follow-up responses by the participants.

A total of 113 participants completed both a baseline and at least one follow-up questionnaire. The number of follow-up responses per participant varied significantly, with a maximum of 53 responses recorded for a single individual. The distribution of follow-up responses (see Fig. 2) highlights substantial variation in participant engagement, a common challenge in longitudinal data collection.

**Fig. 3:**
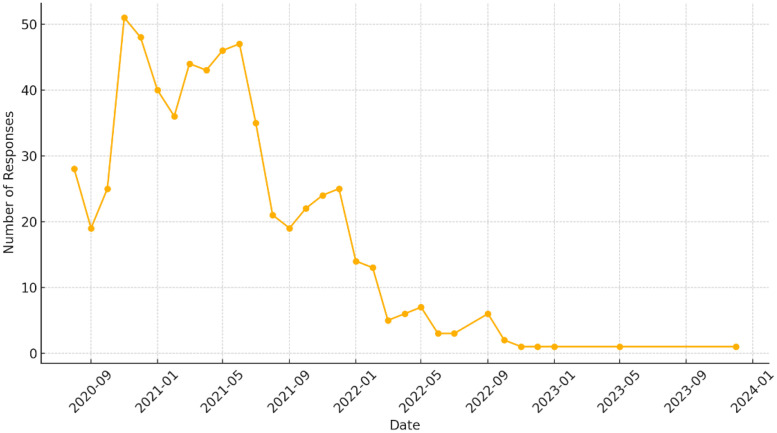
Number of follow-up responses over time.

While the intended interval between follow-up responses was approximately one week, the actual observed average interval was 14.48 days. Such variability in response timing must be carefully considered in subsequent analyses to ensure the robustness of findings. Additionally, participant engagement followed a declining pattern, where a smaller subset of individuals contributed to a disproportionately high number of responses (see Fig. 3). Comparable trends in participant engagement have been observed in previous studies conducted as part of the Corona Health project [8].

Regarding the geospatial distribution of participants within Germany, engagement was highest in North Rhine-Westphalia (55 participants), followed by Bavaria (41) and Baden-Wuerttemberg (25). In contrast, Saxony-Anhalt and Bremen had no participants in the dataset.

This regional variation in response rates aligns with broader trends observed in digital health engagement, where more populous states generally exhibit higher participation. At the same time, the observed attrition effect, where a smaller subset of participants contributed a disproportionately high number of responses, is consistent with findings from other digital health studies [9]. These patterns underscore the importance of understanding both regional engagement disparities and individual participant adherence when interpreting results, as they can significantly influence the representativeness and reliability of longitudinal data.

In general, it is important to clarify the temporal characteristics of our data collection approach. While the Corona Health app utilized EMA methodologies, our implementation featured a lower sampling frequency than traditional high-intensity EMA protocols. The study design initially targeted weekly follow-up assessments to balance data richness with participant retention over the extended study period (July 2020 to December 2023).

This deviation from the intended weekly schedule can be attributed to several factors. First, the voluntary nature of participation meant that adherence was primarily participant-driven rather than enforced through strict prompting mechanisms. Second, the extended duration of the pandemic and associated study period likely contributed to participation fatigue, a common challenge in longitudinal digital health studies. We deliberately chose not to implement overly intrusive notification systems that might have improved adherence but potentially increased participant dropout rates. While this sampling approach provided less temporal granularity than intensive EMA designs, it successfully captured meaningful longitudinal trends in adolescent mental health while maintaining sufficient participant engagement throughout the multi-year study period.

## 4 Experimental Design, Materials and Methods

This section provides an overview of the app’s development, research design, data acquisition process, ethical guidelines, and technical framework.

### 4.1 Application design and implementation

The Corona Health app, developed using the PHP-based TrackYourHealth framework [10], was launched in July 2020 for both Android and iOS devices, offering support in eight languages. Its development involved interdisciplinary collaboration, adherence to regulatory requirements, and integration of established methodologies to ensure high data quality and compliance with ethical standards.

Specialists from various fields, including healthcare professionals and data scientists, worked together to uphold the app’s methodological integrity. It was designed in compliance with EU Medical Device Regulation standards, ensuring strong data protection measures and user safety. To enhance data accuracy, Ecological Momentary Assessment (EMA) techniques were implemented, minimizing recall bias. Additionally, sensor-based and digital phenotyping approaches allowed for the collection of objective data, such as location information, to supplement self-reported responses.

### 4.2 Data acquisition and collection process

The Corona Health app served as a mobile platform for collecting ecological momentary assessments (EMA) and sensor data related to physical and mental well-being during the COVID-19 pandemic. Here's how it functioned from a participant perspective:

After downloading and installing the app, participants first saw a disclaimer requiring consent to proceed. Upon consenting, participants could browse and subscribe to any of the five available studies (mental health for adolescents (data presented in this paper), mental health for adults, physical health for adults, stress recognition, and COMPASS project [1]).

For the study presented at hand, participants completed a one-time baseline questionnaire (15-20 minutes) which established their initial health status. When completing their first questionnaire, participants were asked for permission to collect location data (both Android and iOS users) and app usage statistics (Android users only). Location data was deliberately made coarse-grained (11.1km accuracy) to protect privacy.

**Fig. 4:**
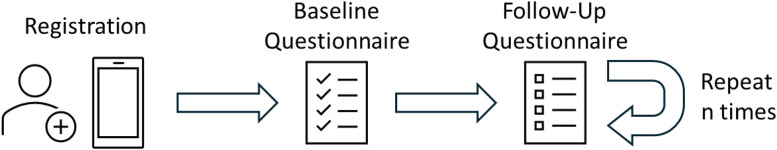
Study Workflow in Corona Health.

Following the baseline assessment, the app scheduled regular follow-up questionnaires (approximately 5-10 minutes) based on each study's protocol (see Fig. 4). Participants received notifications for these follow-ups but could also complete them manually at any time. The responses were stored locally if offline and synchronized with the server when connectivity was available.

The sensor data collected included:

- Device type and operating system information
- Approximate location during questionnaire completion (if permitted)
- For Android users who gave permission: aggregated app usage statistics including daily phone usage, periods of activity/inactivity, and usage statistics for social media apps

All collected data was anonymized and stored in a structured database for research purposes, with feedback provided to participants based on their responses through the app's Info tab.

### 4.3 Survey workflow and data processing

The questionnaire development process began with formulating questions in Microsoft Excel, where metadata, question formats, and language options were defined. These files were subsequently converted into JSON format using Python and integrated into the backend, enabling access through a RESTful API. This approach facilitated dynamic deployment, allowing real-time updates within the app. Data exchange adhered to the JSON protocol, ensuring a structured and efficient process for both questionnaire distribution and response submission.

The app was released in eight languages across Google and iOS app stores. Upon first use, users were presented with a mandatory disclaimer and an onboarding sequence outlining notification preferences and data permissions. The Corona Health system was designed to support both online and offline functionality, ensuring continuous data collection. User data were stored and organized within a relational SQL database, maintaining data integrity and structured management.

## Limitations

A major limitation is the data loss that occurred on December 17, 2020, when an app version update resulted in missing baseline questionnaire responses from 117 participants (29.3% of the sample). While technical metadata (e.g., operating system, GPS data) for these participants remained intact, this data gap impacts the baseline dataset's representativeness and statistical power. However, it's important to note that this data loss affects only the baseline questionnaire and not the follow-up data collection. Researchers using this dataset should approach baseline-dependent analyses with appropriate caution and consider statistical approaches that account for missing data when incorporating baseline variables. Selection bias represents another significant concern, as participation was voluntary and self-selected, likely attracting individuals with higher digital affinity and interest in mental health monitoring. This non-random sampling limits generalizability to broader adolescent populations.

Participant attrition further complicates longitudinal analyses, with only 28.3% of initial participants contributing follow-up data. The highly variable engagement patterns—ranging from single responses to more than 50 follow-ups from individual participants—creates analytical challenges for examining longitudinal trends.

The geographic and temporal inconsistencies in data collection present additional analytical challenges. The regional disparities in participation across German states and the deviation from intended weekly assessment schedules (observed average: 14.48 days) require careful statistical consideration when examining spatial patterns or temporal trends in adolescent mental health outcomes.

## Ethics Statement

This study was conducted in accordance with the German Medical Device Regulations (MDR), the General Data Protection Regulation (GDPR), and the tenets of the Declaration of Helsinki. Data was collected using the Corona Health app. The Ethics Committee and data protection officer of the University of Würzburg, Germany, granted approval for this study (approval no. 130/20-me). All participants provided informed consent for app usage in general, as well as specific consent for GPS-based location data and digital phenotyping metrics. All data were anonymized, and strict measures were implemented to protect participants' privacy throughout the study.

## Data Availability

ZenodoMental Health of Adolescents during COVID-19 (Original data).
